# Privacy Policies of IoT Devices: Collection and Analysis

**DOI:** 10.3390/s22051838

**Published:** 2022-02-25

**Authors:** Mikhail Kuznetsov, Evgenia Novikova, Igor Kotenko, Elena Doynikova

**Affiliations:** 1 Department of Computer Science and Engineering, St. Petersburg Electrotechnical University “LETI”, 197022 St. Petersburg, Russia; mdkuznetsov@stud.eltech.ru (M.K.); novikova@comsec.spb.ru (E.N.); 2 Computer Security Problems Laboratory, St. Petersburg Federal Research Center of the Russian Academy of Sciences, 199178 St. Petersburg, Russia; doynikova@comsec.spb.ru

**Keywords:** privacy policies, dataset, data collection, natural language processing, latent Dirichlet allocation, privacy policy corpus, IoT

## Abstract

Currently, personal data collection and processing are widely used while providing digital services within mobile sensing networks for their operation, personalization, and improvement. Personal data are any data that identifiably describe a person. Legislative and regulatory documents adopted in recent years define the key requirements for the processing of personal data. They are based on the principles of lawfulness, fairness, and transparency of personal data processing. Privacy policies are the only legitimate way to provide information on how the personal data of service and device users is collected, processed, and stored. Therefore, the problem of making privacy policies clear and transparent is extremely important as its solution would allow end users to comprehend the risks associated with personal data processing. Currently, a number of approaches for analyzing privacy policies written in natural language have been proposed. Most of them require a large training dataset of privacy policies. In the paper, we examine the existing corpora of privacy policies available for training, discuss their features and conclude on the need for a new dataset of privacy policies for devices and services of the Internet of Things as a part of mobile sensing networks. The authors develop a new technique for collecting and cleaning such privacy policies. The proposed technique differs from existing ones by the usage of e-commerce platforms as a starting point for document search and enables more targeted collection of the URLs to the IoT device manufacturers’ privacy policies. The software tool implementing this technique was used to collect a new corpus of documents in English containing 592 unique privacy policies. The collected corpus contains mainly privacy policies that are developed for the Internet of Things and reflect the latest legislative requirements. The paper also presents the results of the statistical and semantic analysis of the collected privacy policies. These results could be further used by the researchers when elaborating techniques for analysis of the privacy policies written in natural language targeted to enhance their transparency for the end user.

## 1. Introduction

Currently, IoT devices are actively used not only in critical industries but also in everyday life, creating a comfortable and safe living environment. Such devices are an important part of mobile sensing networks. They collect and process a variety of data, including personal data. The Regulation of the European Union on the Protection of Personal Data—General Data Protection Regulation (GDPR)—defines personal data as data that identifiably describe a person [1]. It also adds to this category data that allow identifying user‘s devices, for example, by their IP and MAC addresses, browser fingerprints, etc. Additionally, it defines the principles of data processing in cross-border data transfer, and the user’s right to erasure. Information about what personal data are collected and processed by devices and/or applications is presented in the privacy policies. However, in major cases, these documents are written in a complex language with a high level of readability. Therefore, users usually do not read them [2,3]. As a result, they give their consent for the collection, processing, and storage of personal data without a clear understanding of how this process is organized, and what risks associated with the data processing arise. At the same time, the consequences of theft and unauthorized use of personal data, especially financial data, health data, and biometric data, can be critical for their owners.

Thus, the problem of making privacy policies transparent that includes the task of increasing the data owners’ awareness about how their data are used is extremely important. Currently, this problem has gained a lot of research interest [4,5,6]. Different approaches that use deep learning techniques to analyze privacy policies have been developed [4,5,6,7,8,9]. They allow one to determine various aspects of personal data usage such as collection, storage, transfer of personal data, etc. The effectiveness of these techniques depends on the quality of annotation of the available datasets and datasets themselves. An analysis of the available datasets and methods of their generation showed that the most common starting point for collecting data is the Amazon Alexa [10].

This service collects statistics on site traffic, and therefore the datasets usually incorporate the most popular sites, including social networking sites, online stores, news resources, etc. Obviously, such services differ from the smart devices in the types of the collected personal data, collection procedures as well as purposes of their use. The smart devices such as fitness trackers, smart watches, smart locks, smart scales, etc., have access to a wider range of personal data, such as the user’s location, biometric data, voice, fingerprints, and facial identification. Thus, in order to improve the accuracy of privacy policy analysis for automated detection of scenarios of personal data used by the IoT devices, it is required to generate a specialized dataset consisting of privacy policies developed specifically for them.

This paper presents a technique for the generation of a dataset of privacy policies for IoT devices and the data collection tool implementing the developed technique. The proposed technique and tool are based on the analysis and parsing of data provided by large e-commerce platforms such as Amazon [11], Walmart [12], and the Google Search service [13]. The generated dataset contains privacy policies for the IoT devices, such as fitness trackers, smart watches, smart locks, smart scales, etc., instead of privacy policies for various websites.

In addition, the authors performed structural and semantic analysis of the generated corpus of privacy policies and conducted its comparative analysis with the existing datasets.

Thus, the main contributions of the paper are as follows:

1. Analysis of the existing corpora of privacy policies with a specification of their key characteristics, such as the year of creation, the number of documents, the collection technique and source, the presence of labeling, etc.
2. The technique for collection of privacy policies specified for the IoT devices.
3. The software tool implementing the developed technique for collection of privacy policies.
4. The corpus of privacy policies specified for the IoT devices and comparative review of its characteristics with other available datasets.
5. Structural and semantic analysis of a generated dataset of privacy policies.

The novelty of the presented results consists of the technique for the dataset generation. The key distinction of the approach lies in the usage of e-commerce platforms as a starting point for the generation of a document corpus. This allows implementing a targeted collection of the links to the IoT device manufacturers’ websites. In addition, the paper presents a comparative analysis of privacy policies outlining their key characteristics, including the presence of labeling.

The paper is organized as follows. Section 2 discusses the related research in the area and provides a comparative analysis of privacy policy datasets. Section 3 describes a developed technique and tool for privacy policies collection. Section 4 outlines the results of the analysis of the generated dataset, including the semantic analysis of the topics presented in the collected documents. Section 5 discusses the results obtained and defines further directions of the research.

## 2. Related Works and Their Comparative Analysis

After the adoption of the GDPR [1] the problem of the analysis of the privacy policies written in natural language has been actively researched. Various methods of structuring and formalizing policies have been developed [5,9,14,15,16]. Different techniques for the analysis of the privacy policies written in natural language including automatic data collection [17], data labeling [17,18,19], personal data usage scenario identification [5,9,20] and calculation of the risk associated with personal data usage [6] have been proposed. For example, in [21,22] authors investigate the problem of the privacy policy ambiguity and generality and propose an ontology-based approach to decrease the generality of privacy policy terms by establishing semantic relations between them. These relations correspond to hypernymy, meronymy, or synonymy relationships.

The most commonly used corpus of privacy policies is the OPP-115 dataset [17]. It was generated as a part of the Usable Privacy Policy (UPP) project [4]. It consists of 115 website privacy policies that were collected using the Amazon Alexa service [10]. The undoubted advantage of this dataset is the presence of annotations and a labeling scheme developed by its authors. It includes various scenarios of personal data usage and information about the experts who annotated the texts. Each policy was labeled by several annotators. It allowed creating over 20,000 annotations reflecting various aspects of personal data usage. In [23], authors defined links between the elaborated annotation scheme and the GDPR principles.

Another annotated corpus of privacy policies is the APP-350 dataset [18]. It consists of the privacy policies posted on the Google Play marketplace [24]. The authors do not provide a detailed description of how it was collected, but it is possible to assume that this dataset was created using the API of Google Play service, which provides wide opportunities for collecting required data. The MAPS dataset [18] is an extension of the APP-350 dataset. It was also generated on the basis of the privacy policies presented in Google Play [24]. However, unlike OPP-115, it is not annotated. This dataset includes over a million privacy policies for Android applications. Though the datasets [17,18] have annotations that describe different aspects of personal data usage, they consist of the privacy policies that were created before the adoption of the GDPR, and, thus, these privacy policies do not consider the requirements of this regulatory document.

In [25], Princeton-Leuven Longitudinal Privacy Policy Dataset [26] is presented. Its distinctive feature is that it consists of more than a million privacy policies that have evolved and changed over the past 20 years so it is possible to track how the privacy policies evolve. The authors discovered that privacy policies have become even more difficult to comprehend over time. In addition, the authors developed a tool that extracts various terms, such as n-grams, named entities, URLs to evaluate how the topics in the privacy policy change over time. The main source of data for the dataset collection was the Amazon Alexa service.

R. N. Zaim and S. Barber [19] presented a dataset of privacy policies in different languages, collected for more than 1.5 million websites. The authors noted that it is quite difficult to find large datasets available in the open access. In their work, the authors used DMOZ, an open large web directory with Internet URLs, containing more than 1.5 million addresses. The authors analyzed the dataset and found out what categories of sites often neglect privacy policies. The authors also considered their dataset to be an excellent tool for researchers who may find it useful to test their analysis techniques on a large corpus of the documents. However, the DMOZ resource is currently invalid and has been replaced by a similar project Curlie that is built on Open Directory Project (ODP) and DMOZ [27].

In [28], a dataset consisting of more than a million privacy policies written in English is described. The authors implemented a series of experiments with this data set to determine the similarity between documents, conducted policy readability tests, extracted aspects of personal data usage scenarios using key phrases and words. They also analyzed the dataset using topic modeling methods.

V. Ahmad et al. generated the “PolicyQA” dataset [8]. This dataset is generated to develop a Question-Answer (QA) system that would allow users to ask questions about the privacy policy and receive accurate and concise answers. This dataset is generated on the basis of the OPP-115 dataset. It consists of 25,017 examples of explanations of privacy policy statements. PolicyQA provides answers to 714 questions generated for various privacy policies.

Another corpus for the development QA systems is presented in [29]. This dataset consists of 1750 questions about the privacy policies of 35 mobile applications. These applications represent different categories of applications available in the Google Play Market. The questions are aligned with the annotation scheme of the OPP-115 dataset.

Table 1 provides a comparative summary of the privacy policy datasets discussed above.

The corpora of privacy policies listed above are actively used in the research projects devoted to the analysis of privacy policies written in natural language. For example, ref. [5] presents the Polisis application, that provides graphical results of privacy policy analysis. Furthermore, the authors of this application developed the PriBot [5] that answers users’ questions about privacy policies in both natural and formalized query languages. It uses machine learning models for its operation. To train them a dataset of 130,000 privacy policies was used. This dataset is not publicly available. The weighted accuracy of identification of various personal data usage scenarios obtained by the authors is 88.4%. The accuracy of obtaining correct answers using the PriBot is 82%.

In [30], the authors developed the PrivaSeer search engine for searching and analyzing privacy policies according to the specified criteria such as readability level, completeness, and accuracy of formulations. This search engine has indexed more than 1.4 million privacy policies.

N. S. Zaim et al. have developed a set of web browser add-ons that simplify the structure of privacy policies [9]. These utilities are based on a statistical analysis of visits to web pages of privacy policies. The authors showed that there is a large number of privacy policies that are regularly visited and read, i.e., the users are interested in privacy policies that provide information on how their data are used.

The semantic framework presented in [16] is designed to formalize privacy policies in the form of ontology. It is based on the OPP-115 dataset [17]. The developers have written 57 SPARQL queries to extract data from an ontological representation to answer questions about privacy policies.

The technique for generating the datasets of privacy policies is described in detail in [20]. The presented crawler works as follows: first, it receives the initial set of root URLs; then it goes through all the URLs that were found, except the duplicates. During data collection, the crawler detects certain labeling patterns and saves data that meet the collection requirement. This tool can be used to collect website privacy policies using the P3P standard [31] because this standard provides a special field in the HTTP protocol header. However, this standard is deprecated, and the majority of websites do not follow this standard, which makes it impossible to use the crawler.

E. Novikova et al. [6] considered the problem of calculating privacy risks based on the analysis of privacy policies. The authors propose an approach that incorporates analysis of the privacy policy written in natural language, generation and automatic processing of the ontology in order to calculate privacy risks associated with a given privacy policy.

In the works discussed above, the main source of data is Amazon Alexa, DMOZ, etc. Such tools and services are not focused on collecting privacy policies of the smart device manufacturers and therefore do not group policies into categories, such as smart device privacy policies.

A distinctive feature of this research is the focus on the collection and analysis of privacy policies developed by the manufacturers of smart IoT devices. These devices are characterized both by a wider range of processed data types and by the ability to receive data from third parties. The necessity to investigate how these aspects are presented in the privacy policies defined a task of generation of a new corpus of specific privacy policies and development of the appropriate techniques and software tools. Thus, the distinctive feature of the crawler presented in this paper is the collection of data from e-commerce platforms under given conditions. The flexible configuration of search parameters of the crawler allows one to select privacy policies that meet the specified requirements. It ensures the relevance of the privacy policies retrieved.

## 3. The Technique for Privacy Policy Corpus Collection

The authors propose to decompose the task of collecting privacy policies into several subtasks. Thus, the technique consists of the following steps:

1. collecting hyperlinks to IoT devices on online store sites;
2. generating a list of manufacturers of IoT devices;
3. searching the websites of manufacturers of IoT devices;
4. searching the privacy policies on the manufacturer’s website;
5. loading privacy policies;
6. sanitizing and preparing privacy policies for further analysis.

**Figure 1:**
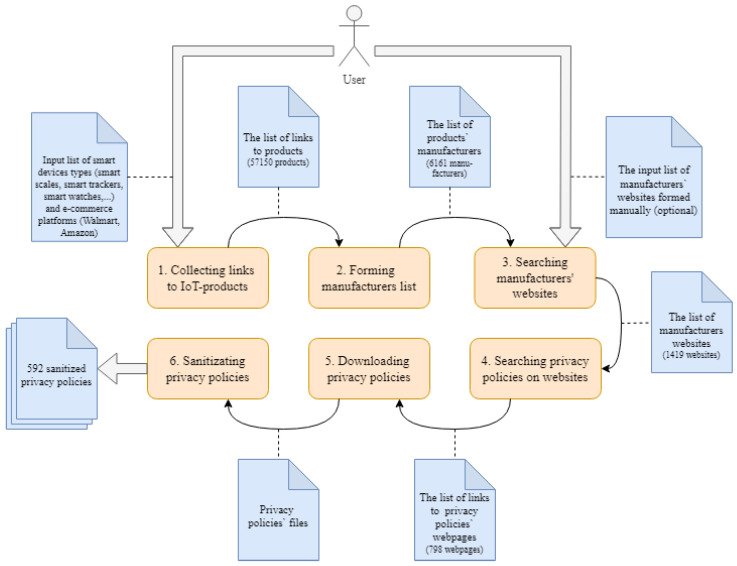
The scheme of the dataset generation technique.

Figure 1 shows these steps in the form of sequentially executing components: the input of one component is the result of the work of the previous component. Thus, the components are connected in one pipeline.

At the first step the crawler collects hyperlinks to the web pages of IoT devices that are available on the e-commerce platforms specified by the user. As the device type is an input parameter, the user can define broader range of devices not just IoT ones. Then, the manufacturer data are retrieved from the page markup. The next step consists of a search of websites of smart device manufacturers. If the manufacturer is found, the search for a link with the privacy policy on the web page is performed. When the hyperlinks to the privacy policies are collected, they are downloaded. After that, the text of privacy policies is sanitized from the HTML markup and code to obtain structured information in natural language. Each of the proposed steps can be implemented in a multithreaded mode since all manipulated objects are not interconnected. Each step is discussed in more detail below.

### 3.1. Collecting Hyperlinks to IoT Devices and Generating a List of Manufacturers

The key difference of the proposed technique is the use of large online e-commerce platforms as a starting point for data collection. The online e-commerce platforms allow one to search for a product by type and receive information about it, including its manufacturer. In general, the online store websites have a typical markup of the web pages that is used then to add and post information about the products. The authors use the Python selenium library [32] to generate the list of URLs for IoT products.

### 3.2. Searching Websites of Smart Device Manufacturers

Usually, the e-commerce platforms do not provide direct links to the official websites of manufacturers. Therefore, in order to obtain the privacy policies of manufacturers of smart devices, it is required to solve the task of searching the official websites of the manufacturers. The search engines such as [33,34,35] provide paid search APIs. There are free analogs, but they have restrictions on the number of requests per month/minute. For example, a lot of these services are hosted on the site [36]. The experiments have shown that the use of search engines designed for real users provides a compromise solution. This is most likely due to the orientation on the end user, i.e., when receiving a query close to the brand name, it is more likely that the search engine will display the given manufacturer’s official website. Thus, the current solution is to construct a list of URLs to manufacturers‘ websites based on the first Google search results page. The URLs are analyzed for similarity with the manufacturer’s name using the built-in Python function to extract the most relevant ones. If the search results do not contain the name of the given manufacturer, then the manufacturer does not have a website and uses an e-commerce platform privacy policy with a probability close to one.

### 3.3. Searching Links to Privacy Policies

Searching for a privacy policy on an already found manufacturer’s website is a quite trivial task. Currently, most websites have a link named “Privacy” or “Privacy Policy” in the footer. The footer is available on any page of the website as it is a part of the global navigation system of a website. It contains information that is not used as often as the data displayed at the top of the sites and in the main menu but is still important. In addition to links to the privacy policy, the website footer often contains contact data of a company or organization.

Collecting privacy policy using the hyperlink in the footer is the subtle point in the proposed technique as this privacy policy could relate to the manufacturer’s website or some generic privacy policy developed by the manufacturer. However, the performed analysis showed that the small companies have privacy policies that are tailored for all purposes, i.e., for a website, for the employees and smart devices. Some companies have two types of privacy policies on one web page: for website and for services and products [37]. In this case the tool collects both privacy policies as one document. Large international companies have a set of privacy policies for different types of services, for example, Huawei has a privacy policy for cloud service, for mobile services, and a generic one for consumers [38]. In this case the collected privacy policy belongs to the manufacturer’s website. The automatic validation of the privacy policy type is included in the scope of future works as it requires a deep analysis of the differences between types of privacy policies developed for different purposes if such exist.

Algorithm 1 represents all steps of the algorithm for privacy policies collecting. Firstly, device types and online stores are read (Algorithm 1, lines 1–2). Then products URLs are found and products manufacturers are extracted (Algorithm 1, lines 3–4). Finally, manufacturers‘ websites are found, crawled to find privacy policies and privacy policies are dowloaded (Algorithm 1, lines 5–7).
**Algorithm 1** Collection of the privacy policiesInput: names_of_marketplaces, device_typesOutput: docs_paths ▹ Path to the directory with downloaded policies in HTML1:marketplaces = read_marketplaces(names_of_marketplaces)2:device_types = read_device_types(device_types)      ▹ Read user input3:urls = find_devices(marketplaces, device_types)4:manufacturers = extract_manufacturers(urls)5:websites = search_websites(manufacturers)6:policies = extract_policies(websites)7:docs_paths = download_policies(websites)8:**return** docs_paths

### 3.4. Privacy Policy Sanitization

Sanitizing the privacy Policies is a complex task. It incorporates several subtasks. First, all tags that are responsible for the interactivity of the web page, including all control elements, are to be removed. The authors also made the following assumptions:

- the GUI elements such as pop-up, modal, and dialog boxes cannot contain the text of the privacy policy;
- the images on the page do not belong to the privacy policy.

For this reason, the corresponding tags are included in the list of tags to be removed.

The next step of document sanitizing involves a restructuring of the web document. It means transforming the HTML markup. The authors elaborated the following HTML markup restructuring rules.

- The generic HTML tags-containers such as “div” are to be expanded.
- A certain combinations of nested tags are to be replaced with simpler ones. For example, the “b” tag nested within the “div”, “span” or “p” tags is replaced with a “strong” tag, an “i” tag nested within a “div”, “span” or “p” tag is replaced with an “em” tag.
- The tags are to be cleared from the attributes since they usually do not contain useful information.

The entire DOM tree of the document is then traversed recursively to merge tags where possible, or to wrap raw text within tags. The final stage of the document sanitization consists of the extraction of the plain text from the tags, as the result, the paragraphs are represented as one long line. This is done because line breaks arranged in a certain way can complicate further analysis including reading. Algorithm 2 represents all steps of privacy policies sanitization.
**Algorithm 2** Common algorithm of privacy policies sanitizing       ▹ This algorithm correspond to step 6 in Figure 11:docs_paths = read_paths()2:filtered_html= remove_tags(docs_paths)3:sanitizer = HtmlSanitizer()     ▹ Use html_sanitizer library4:recombined_html = sanitizer.sanitize(filtered_html)5:extracted_text = extract_policy(recombined_html)

The Listing 1 represents regular expressions and tags, which will be removed during the sanitization process. These tags and regular expressions are used in Algorithms 3 and 4.

**Listing 1.** Regular expressions and tags removed while sanitizing.▹ Uninformative tag types to removetags_to_remove = [ 
“head”, “cart”, “foot”, “nav”, “bar”, “alert”, “modal”, “popup”, 
“banner”, “promo”, “side”, “notify”, “notification”, “toolbar”, “menu”, 
“ft”, “hd”, “navigation”, “shopify”, “footer”, “header” ]
▹ Regular expressions presented as pairs (refexp, substitution)
▹ to implement processing of the HTML tree leaveslocal_exprs = [ (r“[^a-z0-9,.:;\\/\n\s(){}*?![]]”, “”), (r“\n+”, “”), (r“\s+”, “ ”), (r“^[\n ]+”, “”),]
▹ Regular expressions by pairs (refexp, substitution)
▹ to use for HTML text in order to remove all tags,
▹ blank multilines, etc.
global_exprs = [
  (r“[\w\-]+@[a-z]+$\dot{[}$a-z]{2,}”, “{removed e-mail}”),
  (r“(https?://)?(www\.)?(([\w\-]+\.)+[a-z]{2,})(/[\w\-]+)*”, “{removed hyperref}”),
  (r“^\s{4}”, “”),
  (r“([.,:;!?\)\}\]])”, “\g<1>”),
  (r“([.,:;!?\)\}\]])(\w+)”, “\g<1> \g<2>”),
  (r“(\w|[\}\]\)])\n”, “\g<1>.\n”),
  (r“([\[\(\{]) ”, “\g<1>”),
  (r“([\}\]\)])([\{\[\(])”, “\g<1> \g<2>”),
  (r“</?.*/?>”, “”),
  (r“\n{4,}”, “\n\n\n”)
]

Algorithms 3 and 4 represent the sanitization functions in detail. The “remove_tags” function removes all unallowed HTML tags. The “recombine” function alters the DOM structure to make the page code of all policies more unified, clean, and similar. The last function “extract_policy” cuts all the text information from privacy policy HTML tags and puts it into a new text file.
**Algorithm 3** Algorithm of remove_tags function removing all unallowed HTML tagsInput: htmlOutput: modified_html1:modified_html = html                    ▹ Save input html2:peripherals = []                 ▹ Make new list for html nodes3:peripherals.append(modified_html)          ▹ Push html root to the list4:**while** peripheral.length > 0 **do**5:    el = peripheral.shift()                   ▹ Pop first element6:    **for** c in el.children(el) **do**7:        **if** c **then** not in tags_to_remove8:           peripheral.append(c)              ▹ Push children to the list9:        **else**10:           el.remove(c)              ▹ Remove node if it is unallowed11:        **end if**12:    **end for**13:**end while**14:**return** modified_html

**Algorithm 4** Algorithm of extract_policy function cutting all available text in HTML tree
Input: html, local_exprs, global_exprs
Output: policy_text
1:modified_html = html                     ▹ Save input html2:peripherals = []                  ▹ Make new list for html nodes3:peripherals.append(html)               ▹ Push html root to the list4:**while** peripheral.length > 0 **do**5:    el = peripheral.shift()                    ▹ Pop first element6:    **for** c in el.children() **do**7:        **if** type(c) is not leaf **then**       ▹ If element is not a leaf of the tree, skip it8:           peripheral.append(c)         ▹ Add child to the list for future unfolding9:           **continue**10:      **end if**11:      **for** exp in local_exprs **do**12:           c.text = re.sub(c.text, exp)        ▹ Else apply local substitutions to it13:      **end for**14:  **end for**15:
**end while**
16:policy_text = html.to_text()                   ▹ Get policy text17:**for** exp in global_exprs **do**18:    policy = re.sub(policy, exp)      ▹ Apply global substitutions on policy text19:
**end for**
20:**return** policy_text


## 4. The Results of Privacy Policies’ Corpus Analysis

In this section, the authors describe the generated corpus of privacy policies for IoT devices (Section 4.1), the results of its statistical analysis (Section 4.2), and the results of its semantic analysis (Section 4.3).

### 4.1. Description of the Generated Corpus of Privacy Policies for IoT Devices

To create a IoT dataset [39] the search was implemented the Amazon [11] and Walmart [12] e-commerce platforms. The authors considered the following types of smart devices: smart scale, smart watch, smart bracelet, smart lock, smart bulb, smart navigation system, smart alarm clock, smart thermostat, smart plug, smart light switch, smart TV, smart speaker, a smart thermometer, smart air conditioner, smart video doorbell, robot vacuum cleaner, smart air purifier, GPS tracker, tracking sensor, indoor camera, outdoor camera, and voice controller. The search query results for the first thirty pages were analyzed.

**Figure 2:**
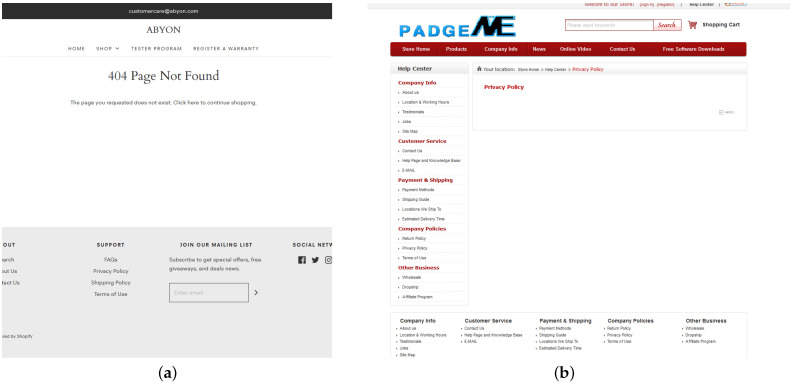
Examples of websites with missing policies: (**a**) the link to the page with the privacy policy exists, but there is no page with the text of the document; (**b**) the page with the privacy policy exists, but it is empty.

The authors analyzed 57,150 smart product models in total, and 51,727 manufacturers were identified for them, that is 90.5% of all collected devices, and 6161 unique manufacturers among them. There are 1419 (23%) of manufacturers that have an official website. When processing official websites of the manufacturers only 798 English-language privacy policies were obtained. The authors also excluded privacy policies, which length in characters did not exceed 1000. The manual analysis of such documents revealed, that they appear either due to the fact that some manufacturers have an empty page with a privacy policy on their website or do not have it at all. Figure 2 shows examples of such privacy policies. Thus, the number of the collected unique privacy policies is 592.

The authors consider this result as quite good since many manufacturers do not have their own websites. In these cases, the privacy policy of the online e-commerce platform is used for the device, for example, Amazon’s privacy policy. It should be noted that in this case privacy policies for the same device may differ due to different regulatory requirements adopted in the country in which the products are shipped.

In some cases, a product’s manufacturer is not specified explicitly. Such devices were also excluded from the collection. The authors did not detect the cases when one manufacturer developed different privacy policies for different types of manufactured devices. Though this situation is possible but requires elaboration of another approach to privacy policy collection, such an approach should include the step of the analysis of the product documentation.

### 4.2. Statistical Characteristics of the Privacy Policy Corpus

To define techniques for further processing and analysis of privacy policies, it is required to implement a preliminary statistical analysis of the collected corpus. The authors analysed the length of the documents in the whole, paragraphs, the number of paragraphs, and other methods of data structuring in the text.

**Figure 3:**
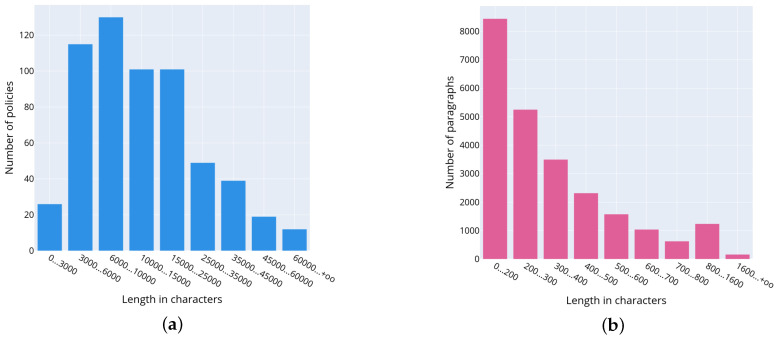
Characteristics of privacy policies of the corpus: (**a**) distribution of privacy policies by length in characters; (**b**) distribution of lengths of privacy policy paragraphs in characters.

Figure 3a presents the distribution of policies by their length in characters. To construct it, the authors evaluated the length of the policies in characters and number of the documents. Formally, the calculation procedure is described as follows. Let $X={\left\{x_{i}\right\}}_{i=0}^{k-1}$ be a set of documents‘ lengths, where $x_{i}$ is the length of *i*-th document, and *k* is a number of documents. Let $A={\left\{\left[a_{j},a_{j+1}\right)\right\}}_{j=0}^{n}$ be a set of bins used to construct a histogram. The size of bin is a variable. Let $1_{\left\{x_{j}\in \;\left[a_{i},a_{i=1}\right)\right\}}$ be an indicator function that returns true if $x_{j}$ is in the given interval $\left[a_{i},a_{i+1}\right)$. Let $n_{i}$ is a counter of the documents that have length in characters in the interval $\left[a_{i},a_{i+1}\right)$, then $n_{i}$ is determined as $n_{i}=\sum_{j=0}^{k-1}1_{\left\{x_{j}\;\in \;\left[a_{i-1},a_{i}\right)\right\}},i=1,\ldots ,k$. The plot visualizes $n_{i}$ values for each histogram bin.

It can be noticed that the most common document length is 6000–10,000 characters. It corresponds to 3.3–5.5 standard pages of 1800 characters. Figure 3b shows the distribution of privacy policy paragraphs by the length in characters. This plot is constructed in the way similar to the plot in Figure 3a. Obviously, the most common are short paragraphs up to 200 characters long consisting of 2–3 sentences.

The distribution of text structural elements such as tables, lists in the corpus of privacy policies, was also analyzed. The understanding of how the text in privacy policies is organized helps to define algorithms and procedures for its further analysis and correct extraction of the text excerpts describing the personal data usage scenarios.

**Figure 4:**
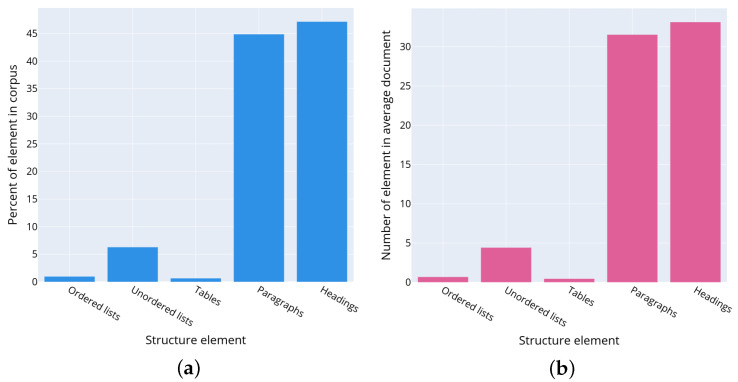
Characteristics of privacy policies of the corpus: (**a**) distribution of the text structural elements in the text of privacy policy; (**b**) number of the text structural elements in the text of average privacy policy.

It was discovered that it is rather difficult to automate counting the number of headings due to the wide variety of HTML markup used to generate them. Almost all websites use their own HTML markup, their own convention for numbering sections, headings, and lists. For example, on some websites, lists and headings are numbered using HTML, on others, the numbering is set manually. In addition, the implemented analysis demonstrated that privacy policy headings can be generated differently even across multiple privacy policies belonging to one manufacturer. For example, Huawei developed about 50 privacy policies for various services [38]. Although they all have the same structure visually, up to 16 different patterns of HTML markup tags are used for headings. Thus, even within one company, there is no unified convention for the privacy policies design and its structural layout. The authors suggest considering as headings the strings less than 100 characters long and not containing the “list item” markers (list item marker). However, this approach does not give accurate results as the short paragraphs consisting of one line such as the contact information of manufacturers are also referred to as headings. The distribution of text structural elements in the policies corpus is represented in Figure 4a. The plot is constructed as follows. Let $S={\left\{s_{i}\right\}}_{i=0}^{n-1}$ be a set of text structural elements $s_{i}$, where *n* is a number of structural elements in the corpus. Let $T={\left\{t_{j}\right\}}_{j=0}^{4}$ be a set of types such as ordered list, unordered list, tables, paragraphs, and headings. Let function type returns a type of the text structural element, then $G_{t}=\left\{x_{k},type\left(x_{k}\right)=t,t\in T\right\}$ is a set of structural elements $x_{k}$ of the given type *t*. The number $n_{t}$ that characterizes the ratio of the text structural elements of the given type in the dataset is determined as $n_{t}=\frac{|G_{t}|}{\left|X\right|}*100,$ where || denotes to the set capacity, i.e., a number of elements in the set. The plot visualizes $n_{t}$ values for each type of the text structural element.

Figure 4b shows the statistically average privacy policy, which can be described as a document consisting of 31.5 paragraphs (44.9% of the document), 33 headings (47.2% of the document), 0.7 numbered lists (1% of the document), 4.4 unnumbered lists (6.3% of the document), and 0.5 Tables (0.7% of the document).

**Figure 5:**
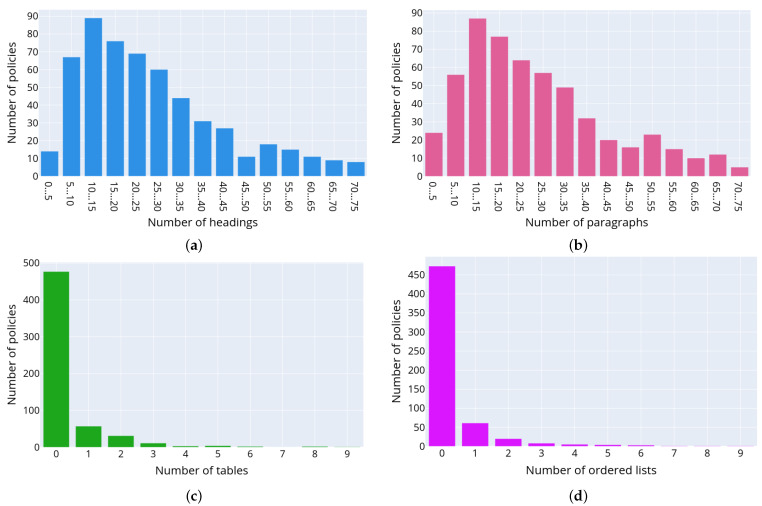

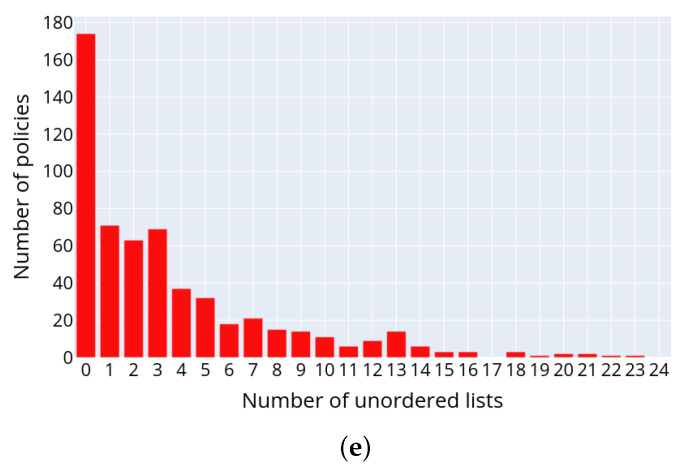
Histogram of the distribution of data structuring elements in the text of privacy policies: headings (**a**), paragraphs (**b**), tables (**c**), numbered lists (**d**) and unnumbered lists (**e**).

**Figure 6:**
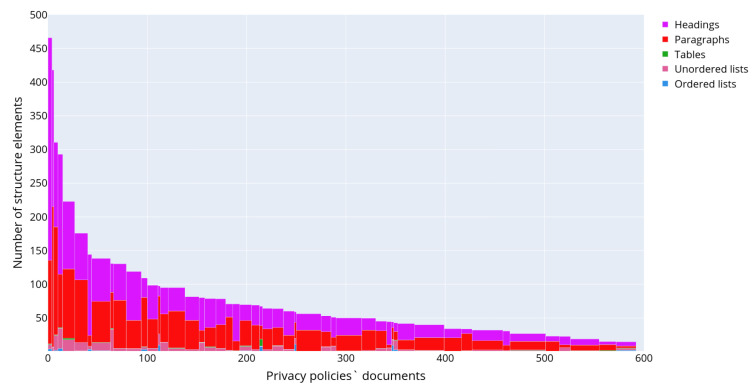
Structure of the privacy policies in corpus “IoT dataset”.

Figure 5 depicts the distributions of the following text structural elements in the privacy policies: headings (a), paragraphs (b), tables (c), numbered lists (d) and unnumbered lists (e), respectively. The presented data allow concluding on the structure of privacy policies. Thus, usually privacy policies consist of headings and paragraphs. The most commonly used structure elements are numbered or unnumbered lists and their elements, while the tables are used rarely. Figure 6 outlines a detailed distribution of the text structural elements for each of the retrieved privacy policies. The documents are ordered and grouped by the number of text structured elements and are displayed along the *x*-axis, while the *y*-axis indicates the total number of structural elements in the text of each privacy policy. The color reflects the type of text structural element.

**Figure 7:**
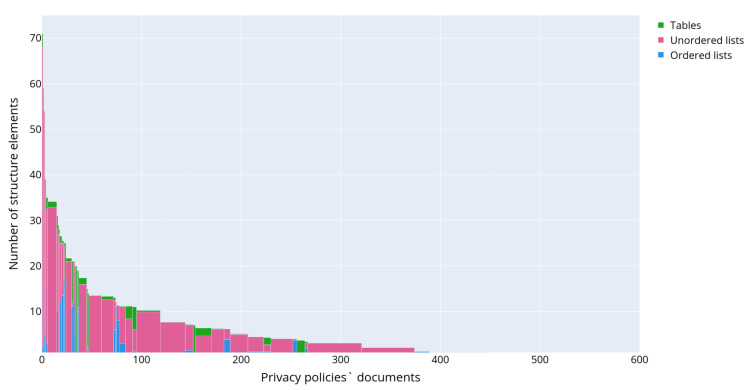
The distribution of the text structural elements excluding headings and paragraphs in the privacy policies in corpus “IoT dataset”.

Figure 7 shows the distribution of text structural elements, excluding headings and paragraphs, as they are present in each privacy policy.

### 4.3. Semantic Analysis of the Generated Corpus of Privacy Policies

To assess the presence of the different aspects of personal data usage in the corpus of privacy policies, the latent Dirichlet allocation method (LDA) was used. It allows representing a document as a combination of topics [40]. In order to apply this method, each policy was represented as a set of paragraphs, based on the assumption that each paragraph may contain a description of one or more personal data usage scenarios. Thus, the implemented analysis process included the following steps:

1. Extracting text paragraphs from documents.
2. Generating topic models based on the analysis of the entire set of paragraphs.
3. Determining the personal data usage scenarios according to the obtained semantic models.

Before applying LDA, each paragraph was transformed into a vector of words. Then the stop words, i.e., the most frequent words that are not significant for the meaning, were removed. Then the text was lemmatized using Python NLTK library [41] in order to generalize some words and achieve more accurate results. Experiments described in [5] have shown that the TF-IDF metric allows extracting more detailed information about the data usage scenarios since this text vectorizing model assigns higher weights to words that are less common. Accordingly, it allows identifying some nuances of the personal data usage scenario more accurately.

The optimal number of semantic models of topics presented in the corpus of privacy policies was determined as 15 since it corresponds to the maximum value of coherence calculated using Gensim library [42].

Table 2 provides the results of topic modeling in the generated privacy policy corpus for IoT devices. The semantic space coordinates column shows the coordinates of the semantic space, given as keywords and arranged in descending order by their weights.

Possible data scenarios were determined manually by authors based on the analysis of the extracted keywords used as coordinates of the semantic space. The authors tried to be close to the 11 personal usage scenarios identified in [18]. These scenarios include such aspects as the first party collection and usage, the third party sharing, data retention, data security, user controls, opt-in/opt-out, do not track, international and special audience, and so on. It was not a trivial task as the topic could be assigned to several scenarios simultaneously or it was not obvious as in cases of topics 4 and 12. However, the authors noticed that semantic clustering allows distinguishing various aspects of privacy policies.

**Figure 8:**
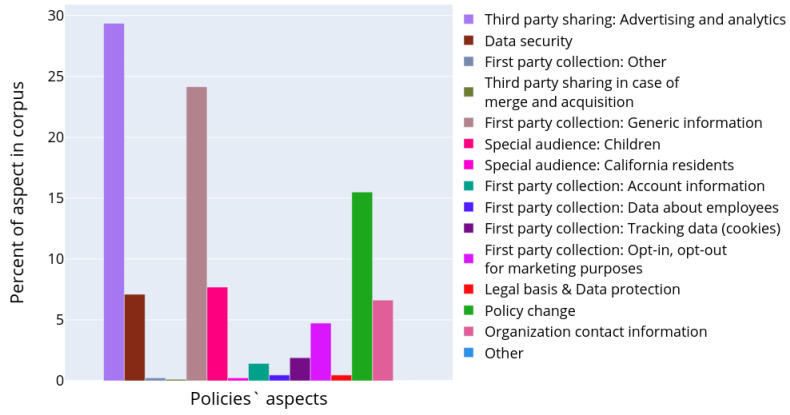
Distribution of various topics describing personal data usage scenarios in the collected corpus of privacy policies.

Figure 8 depicts the results of semantic analysis for the IoT dataset corpus of documents. One paragraph could be attributed to several topics as soon as it was demonstrated that paragraphs may contain different aspects of personal data usage [7]. The paragraphs were assigned to the corresponding clusters with an affiliation probability of more than 70%, in the rest cases they were omitted from the analysis. The chart in Figure 8 characterizes the occurrence of the topic in the entire corpus of documents. It reflects what part of all text volume is devoted to certain aspects of personal data usage. The bar plot was constructed in a manner similar to the plot in Figure 4, except the authors calculated the considered paragraphs only and topic models instead of text structural elements and their types, correspondingly.

It is clearly seen that particular attention in privacy policies is made on how the data sharing with third parties is performed. The information about first party collection is also widely presented, though it is clearly seen that many paragraphs still provide generic information. However, the aspect of opt-in/opt-out in marketing purposes is discussed. The privacy policies also contain enough information on how the end users are notified in case of policy change. Some topics were quite unexpected, for example, there are paragraphs devoted to the data belonging to the potential employees in the privacy policies (topic 6 in Table 2). This information seems to be irrelevant to the privacy policy designed for the smart devices, which should be focused on a user of the product and cover the aspects of their personal data collection and processing. However, the presence of such information could be explained by the attempt of the manufacturer to design one unified privacy policy for all cases and purposes.

**Figure 9:**
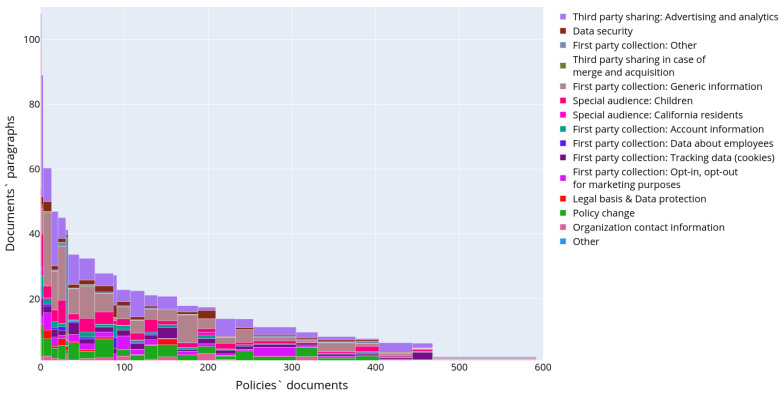
Distribution of the topics in the text of the privacy policies in IoT dataset.

Figure 9 shows the distribution of the semantic topics in privacy policies in detail. The topic affiliation threshold was set to 70%. To construct it, the documents were firstly divided into clusters according to the topic distribution in their texts. This was done using the k-means clustering method. Each privacy policy is represented with a vector containing numbers of elements of a certain type. Then the privacy policies were ordered by cluster firstly and then by the number of paragraphs in the document. Thus, X-axis corresponds to the document number in the ordered list of all documents, and, therefore, the width of the histogram bar is proportional to the quantity of the documents in the corresponding cluster. The Y-axis in Figure 9 shows the centroid coordinates of the cluster, i.e., numbers of paragraphs assigned to each topic model. In other words, it shows the average number of paragraphs in the document cluster, and colored sections of the bar reflect the number and ratio of topics in each specific cluster. Thus, it is possible to conclude that all topics characterizing aspects of personal data usage are widely presented only in an extremely small number of documents. These documents constitute the first three clusters-outliers in terms of the number of paragraphs. These clusters contain 1, 2, and 10 policies, correspondingly. The possible reason for the existence of such clusters-outliers is the duality and vagueness of these privacy policies when some paragraphs are assigned to several topics simultaneously with high affiliation probability. In general case the majority of the documents contains a small number of different semantic topics. The most widely covered topic devoted to “Third party sharing” (topic 14 in Table 2).

To understand if there is any semantic difference in the texts of the privacy policies collected by the developed tool and other existing data sets, the authors performed a similar semantic analysis with similar experiment settings with the popular OPP-115 dataset [17]. The privacy policies from the OPP-115 dataset were preprocessed in the similar manner, and a results of the topic modeling are presented in Table 3. Similarly, the semantic space coordinates column shows the list of the semantic space coordinates presented by the keywords and arranged in descending order by their weights. The possible usage scenario was determined on the basis of these keywords. These usage scenarios also slightly differ from the ones defined in [18] as the authors added some additional specifications of their aspects.

**Figure 10:**
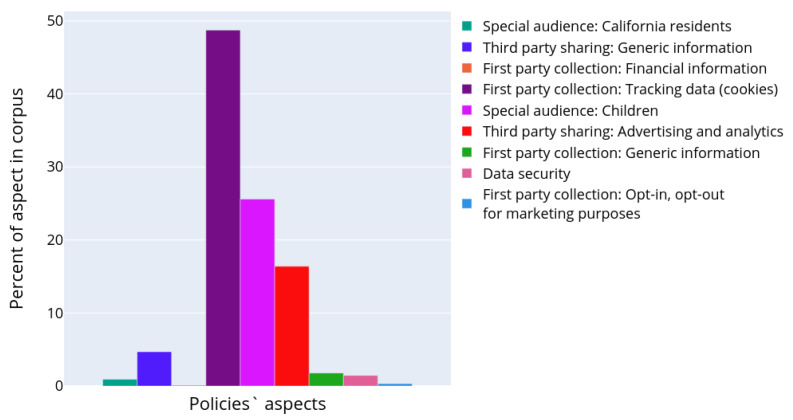
Distribution of various topics describing personal data usage scenarios in the OPP-115 corpus of documents.

Figure 10 shows how these topics are represented across the whole corpus of the privacy policies. Similar to the previous experiments, one paragraph could be attributed to several topics simultaneously, since paragraphs may describe different aspects of personal data usage. The paragraphs were assigned to the corresponding topic with an affiliation probability of more than 70%. Figure 10 shows what part of all text volume is devoted to some topic (or aspect of personal data usage scenario). It is clearly seen that the most frequently presented topics relate to the first party collection of tracking data (topics 11 in Table 3), third party sharing in marketing and advertising purposes with opt-out possibilities (topics 0, 4, 5, and 6 in Table 3).

The aspects relating to a special audience are also presented quite wide. The rest topics are presented much more rarely. When comparing the topics of the OPP-115 dataset with topics of the IoT dataset, it is obvious to see that they are not so diverse. Furthermore, this fact is explained that the semantic space coordinates are quite generic and uncertain, and it was not possible to extract some specific features of the personal data scenarios in order to distinguish between topics.

**Figure 11:**
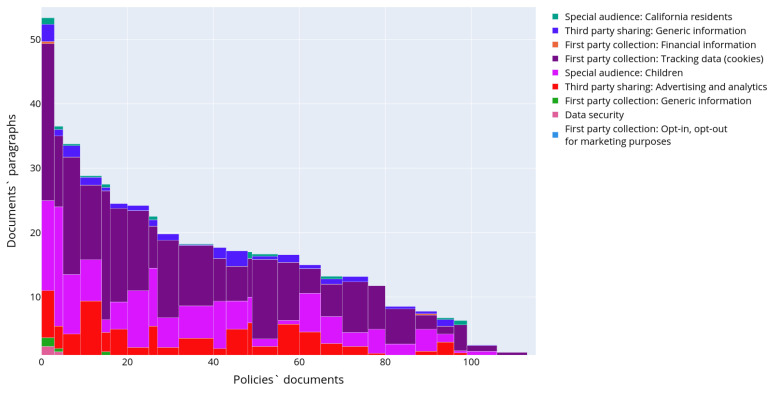
Distribution of the topics in the text of the privacy policies in the OPP-115 dataset.

Figure 11 shows a distribution of semantic topics in the privacy policies of the OPP-115 dataset in detail. The affiliation threshold was set to 70%. The key difference between the chart in Figure 11 and the chart in Figure 9 is that there are no noticeable outlying clusters. However, it should be noted that the number of the recognized paragraphs in policies was less than in the IoT dataset. Based on the distribution of topics across the documents it is clearly seen that the major focus of the privacy policies is the first party collection, but the level of the details of these topics is not high, the semantic space coordinates contain quite generic keywords like “account”, “personal” without detailing what types of data are used.

Thus, when comparing the semantic topics of the IoT dataset and the topics of the OPP-115 dataset it is clearly seen that the IoT dataset topics are more diverse, even if they relate to the first party collection usage scenario. It is possible to differentiate topics relating to financial data collection, tracking data as well as data about employees. The purposes of the data collection are more clearly presented. There are separate topics relating the third party sharing including the case of merger and acquisition, while semantic topics of the OPP-115 are limited to the third party sharing for marketing purposes only. Some topics of the IoT dataset reveal new aspects of personal data processing. They relate privacy policy change, the opt-in/opt-out possibilities provided to the user are presented more widely. These changes in the semantic topics are explained by the requirements of the GDPR [1], which was adopted after the creation of the OPP-115. Moreover, the privacy policies of the IoT dataset contain a larger number of different semantic topics that makes this dataset more suitable for training analysis models to detect different aspects of personal data usage scenarios or reason about them.

## 5. Conclusions

Privacy policies are an official way to inform users what personal data are collected and how these data are processed. Usually these policies are hard to read and understand. As a result, users skip them and do not realize who and how use their personal data and what are their privacy risks. Thus, the task of automated analysis of privacy policies written in natural language and their representation in transparent form is highly relevant. It is especially important nowadays because of the requirements of the regulatory documents to the transparency of the personal data processing, on the one hand, and the rapid development of the Internet of Things, on the other hand. Every day people use a lot of smart devices that collect a large variety of personal data including sensitive ones such as health and biometric data and do not consider related privacy risks.

Currently, the researchers proposed different machine learning based approaches to analyse the privacy policies written in natural language and represent them in transparent form. Application of such approaches requires the usage of annotated datasets of privacy policies to train analysis model to extract specific data collection and usage scenarios. The authors of this paper conducted a comparative analysis of existing datasets and outlined their distinctive features, such as the year of creation, volume, and the presence of labeling. The analysis showed that the available datasets mainly contain the privacy policies of the websites collected from the Amazon Alexa service, and there is a certain lack of the datasets of privacy policies designed specifically for the IoT devices. The analysis of datasets also showed that the currently available datasets usually contain rather old policies collected before the GDPR adoption. Such policies do not contain some scenarios of data collection and processing. Thus, generating and analysis of a dataset incorporating modern privacy policies for IoT devices is a highly important task for further development of new techniques for the analysis of the privacy policies written in natural language.

The authors developed the original technique for collecting privacy policies of IoT devices and the tool implementing the developed technique [43]. The technique uses e-commerce platforms as a starting point for generating a document corpus, while the existing approaches utilize the Amazon Alexa service [10]. It uses the e-commerce platforms to implement a targeted collection of the hyperlinks to the manufacturers’ websites and then uses the collected hyperlinks to download privacy policies of smart IoT devices.

The tool allows specification of the e-commerce platform to be processed and types of devices to be analyzed. The researchers can use the developed tool to collect their own datasets based on their requirements to the platform and device types or use the dataset collected by the authors. However, the developed technique has some limitations as it does not consider the cases when a manufacturer has a set of different privacy policies for different purposes. This issue will be researched in future work.

The authors used the developed tool to collect the corpus of documents [39]. The authors analyzed 57,150 smart product models and identified 51,727 manufacturers (90.5% of all collected devices). There were only 6161 unique manufacturers among them. 1419 manufacturers (23%) have an official website. The authors excluded not English-language privacy policies and privacy policies, which length in characters is less than 1000. Finally, the collected dataset contains 592 unique privacy policies of different IoT device manufacturers. Compared to the existing datasets, the collected dataset contains versions of policies created after the adoption of the GDPR. It bridges the gap between a need for a new dataset of privacy policies for devices and services of the Internet of Things and the absence of such datasets. The provided dataset can be used by the researchers to develop machine learning based approaches for the privacy policies analysis and representation in a transparent form.

The paper presents the main characteristics of the generated corpus of documents, including the results of semantic modeling. Though, the results of the structural and semantic analysis are not valuable for the IoT consumers in a direct manner, however, they are useful for the researchers when elaborating techniques for further analysis of the privacy policies written in natural language targeted to enhance their transparency to the end users. Thus, the semantic analysis revealed significant changes in the topics presented in the generated dataset in comparison with the popular OPP-115 dataset that was created before the GDPR adoption. For example, topics relating to privacy policy change and the right to request access to data have appeared. The latter means that when used for training analysis models with appropriate annotations this dataset could increase the accuracy of detection and reasoning about different aspects of personal data usage scenarios including aspects relating to user control and obligations of the data processors such as the right to access the data and notification in case of policy change.

Future research will include the generation of a similar datasets of policies in other languages, the elaboration of the automatic validation of the collected documents, the development of a document labeling scheme for dataset labeling, further automatic detection of various aspects of personal data usage, and the calculation of privacy risks associated with the use of a device or service.

## Figures and Tables

**Table 1:** Comparative analysis of privacy policies’ datasets.

No.	Naming	Number of Elements	Creation Date	Policies’ Creation Date	Data Source	Annotation	Purpose and Features
1	OPP-115 [17]	115	2016	Until 2018 (policy versions at the time of collection: April to May 2018)	Amazon Alexa	Yes	Policy research under the Usable Privacy Policy Project. Annotated by qualified lawyers, own annotation method.
2	MAPS [18]	>1,000,000	2019		Google Play	No	
3	APP-350 [18]	350	2019		MAPS	Yes	
4	Princeton-Leuven Longitudinal Privacy Policy Dataset [25]	>1,000,000	2021	2001–2021	Amazon Alexa	No	Designed for evaluating changes in privacy policies over time. Contains policies for the last 20 years, the authors also presented a crawler.
5	A Large Publicly Available Corpus of Website Privacy Policies Based on DMOZ [19]	>1,000,000	2020	Until 2017	DMOZ	No	Generation of a dataset for further research. For the generation DMOZ, the largest network directory, was used.
6	PrivaSeer: Corpus of Web Privacy Policies [28]	>1,000,000	2020	Until 2020	Common Crawl data collected since 2008. It contains raw web page data, extracted metadata and text extractions	No	Part of the PrivaSeer, a privacy policy search engine project. The own technique for generating the dataset is developed, it includes a crawler, filters, classification methods, and deduplication.
7	PolicyQA: A Reading Comprehension Dataset for Privacy Policies [8]	25,017	2020	Until 2018	OPP-115	Yes	Part of the PrivacyCheck project. Consists of 25,017 examples of privacy policy language explanations, provides answers to 714 privacy policy questions.
7	PRIVACYQA [29]	1750	2019	Until April 2018	Google Play	Yes	Consists of 1750 questions for 35 privacy policies in English. The questions represent categories of OPP-115 labeling scheme.
9	IoT dataset (this work)	592	2021	Latest versions at the time of creation	Amazon, Walmart, Google, and IoT devices manufacturers websites	No	Designed for analysis of the privacy policies written for smart devices. Generated based on the privacy policies of IoT device manufacturers.

**Table 2:** Results of topic modeling for the IoT dataset.

No.	Semantic Space Coordinates	Possible Usage Scenarios
0	0.029 × privacy + 0.026 × policy + 0.019 × question + 0.013 × link + 0.013 × website + 0.013 × contact + 0.012 × u + 0.011 × practice + 0.010 × third + 0.010 × please	Organization contact information
1	0.023 × privacy + 0.022 × policy + 0.018 × change + 0.012 × time + 0.011 × right + 0.010 × data + 0.010 × update + 0.010 × term + 0.009 × california + 0.009 × personal	Policy change
2	0.036 × gdpr + 0.030 × 6 + 0.017 × 1 + 0.015 × fraud + 0.014 × f + 0.013 × data + 0.013 × b + 0.012 × right + 0.012 × protection + 0.012 × complaint	Legal basis & Data protection
3	0.022 × email + 0.015 × send + 0.014 × opt + 0.012 × product + 0.012 × communication + 0.011 × marketing + 0.010 × may + 0.010 × unsubscribe + 0.010 × u + 0.010 × promotion	First party collection: Opt-in, opt-out for marketing purposes
4	0.025 × insert + 0.023 × para + 0.021 × digital + 0.018 × 2 + 0.014 × 3 + 0.014 × alliance + 0.011 × portal + 0.011 × opt + 0.010 × granted + 0.010 × free	Other
5	0.045 × cookie + 0.020 × browser + 0.016 × technology + 0.015 × device + 0.015 × website + 0.013 × cookie + 0.011 × file + 0.011 × tracking + 0.011 × site + 0.011 × visit	First party collection: Tracking data (cookies)
6	0.020 × location + 0.011 × password + 0.010 × device + 0.009 × professional + 0.009 × sign + 0.008 × employment + 0.008 × account + 0.008 × withdrawal + 0.007 × optimize + 0.007 × notification	First party collection: Data about employees
7	0.032 × address + 0.021 × name + 0.021 × number + 0.015 × purchase + 0.015 × phone + 0.015 × email + 0.011 × inquiry + 0.010 × payment + 0.010 × telephone + 0.009 × contact	First party collection: Account information
8	0.023 × 7 + 0.011 × feedback + 0.010 × california + 0.009 × 18 + 0.009 × evaluate + 0.008 × sufficient + 0.008 × discretion + 0.008 × resident + 0.008 × anyone + 0.008 × meaning	Special audience: California residents
9	0.017 × law + 0.014 × pii + 0.013 × request + 0.012 × personal + 0.012 × child + 0.011 × right + 0.011 × comply + 0.009 × information + 0.008 × applicable + 0.008 × legal	Special audience: Children
10	0.028 × personal + 0.025 × purpose + 0.023 × information + 0.023 × category + 0.023 × collect + 0.022 × data + 0.015 × collected + 0.015 × use + 0.015 × following + 0.013 × processing	First party collection: Generic information
11	0.013 × social + 0.013 × medium + 0.012 × asset + 0.010 × caloppa + 0.010 × acquisition + 0.010 × hesitate + 0.010 × operates + 0.009 × transfer + 0.009 × merger + 008 × require	Third party sharing in case of merge and acquisition
12	0.022 × de + 0.020 × sell + 0.012 × party + 0.012 × property + 0.011 × behavioral + 0.010 × price + 0.010 × disclose + 0.009 × purchased + 0.009 × obtained + 0.008 × defend	First party collection: Other
13	0.022 × security + 0.016 × data + 0.012 × state + 0.011 × united + 0.011 × personal + 0.009 × card + 0.009 × transmission + 0.008 × payment + 0.008 × transfer + 0.008 × country	Data security
14	0.017 × service + 0.016 × google + 0.015 × advertising + 0.014 × website + 0.014 × use + 0.013 × third + 0.012 × party + 0.012 × product + 0.011 × information + 0.011 × analytics	Third party sharing: Advertising and analytics

**Table 3:** Results of topic modeling for the OPP-115 dataset.

No.	Semantic Space Coordinates	Possible Usage Scenarios
0	0.007 × communication + 0.006 × receipt + 0.006 × device + 0.005 × party + 0.005 × third + 0.005 × email + 0.005 × opt + 0.004 × promotional + 0.004 × personal + 0.004 × instruction	First party collection: Opt-in, opt-out for marketing purposes
1	0.009 × healthcare + 0.005 × secure + 0.005 × security + 0.005 × social + 0.005 × opt + 0.005 × mail + 0.005 × network + 0.004 × data + 0.004 × personal + 0.004 × successor	Data security
2	0.008 × collect + 0.006 × job + 0.004 × identifiable + 0.004 × site + 0.004 × personally + 0.004 × cancel + 0.003 × service + 0.003 × information + 0.003 × passive + 0.003 × use	First party collection: Generic information
3	0.024 × account + 0.005 × policy + 0.005 × personal + 0.005 × collect + 0.005 × privacy + 0.005 × service + 0.005 × website + 0.005 × question + 0.004 × use + 0.004 × site	First party collection: Generic information
4	0.005 × personal + 0.004 × service + 0.004 × health + 0.004 × party + 0.004 × conversation + 0.004 × third + 0.004 × poll + 0.004 × promotion + 0.003 × policy + 0.003 × linked	Third party sharing: Advertising and analytics
5	0.009 × cookie + 0.007 × site + 0.007 × service + 0.006 × website + 0.006 × web + 0.006 × use + 0.006 × personal + 0.005 × third + 0.005 × advertising + 0.005 × party	Third party sharing: Advertising and analytics
6	0.006 × party + 0.006 × third + 0.006 × cookie + 0.006 × share + 0.006 × service + 0.005 × personal + 0.005 × may + 0.005 × advertising + 0.005 × website + 0.005 × site	Third party sharing: Advertising and analytics
7	0.008 × child + 0.007 × email + 0.006 × personal + 0.006 × u + 0.006 × service + 0.006 × website + 0.006 × collect + 0.005 × user + 0.005 × communication + 0.005 × information	Special audience: Children
8	0.007 × website + 0.006 × child + 0.005 × collect + 0.005 × address + 0.005 × visit + 0.005 × party + 0.005 × third + 0.004 × site + 0.004 × identifiable + 0.004 × privacy	Special audience: Children
9	0.008 × cookie + 0.008 × personal + 0.007 × website + 0.006 × service + 0.006 × use + 0.006 × site + 0.006 × address + 0.006 × u + 0.005 × policy + 0.005 × email	First party collection: Tracking data (cookies)
10	0.014 × cookie + 0.008 × flash + 0.006 × browser + 0.006 × collect + 0.005 × data + 0.005 × device + 0.005 × user + 0.005 × personal + 0.005 × account + 0.005 × service	First party collection: Tracking data (cookies)
11	0.006 × ticket + 0.005 × mastercard + 0.005 × onto + 0.005 × instruct + 0.004 × obligation + 0.003 × active + 0.003 × personal + 0.003 × long + 0.003 × licensors + 0.003 × retain	First party collection: Financial information
12	0.007 × service + 0.006 × site + 0.006 × data + 0.005 × party + 0.005 × security + 0.005 × third + 0.004 × personal + 0.004 × may + 0.004 × policy + 0.004 × provider	Third party sharing: Generic information
13	0.010 × resume + 0.009 × gameplay + 0.006 × game + 0.005 × third + 0.005 × party + 0.005 × collect + 0.005 × website + 0.004 × social + 0.004 × type + 0.004 × beacon	Third party sharing: Generic information
14	0.007 × california + 0.007 × change + 0.006 × truste + 0.006 × privacy + 0.005 × policy + 0.005 × request + 0.005 × u + 0.005 × contact + 0.005 × email + 0.004 × personal	Special audience: California residents

## Data Availability

The dataset created in this article can be found on github: https://github.com/user160244980349/iot-dataset (accessed on 30 January 2022). The software tool for crawling and sanitization of privacy policy documents can also be found on github: https://github.com/user160244980349/iot-crawler (accessed on 30 January 2022).

## Abbreviations

The following abbreviations are used in this manuscript:

API	Application Programming Interface
DOM	Document Object Model
GDPR	General Data Protection Regulation
GUI	Graphical User Interface
GPS	Global Positioning System
HTML	HyperText Markup Language
HTTP	HyperText Transfer Protocol
IoT	Internet of Things
IP	Internet Protocol address
MAC	Media Access Control address
NLTK	Natural Language Toolkit
P3P	Platform for Privacy Preferences
SPARQL	SPARQL Protocol and RDF Query Language
TF-IDF	Term Frequency–Inverse Document Frequency metric
URL	Uniform Resource Locator
